# Practice preferences of pre-graduation allied health professionals: do graduates want to work where the workforce is needed?

**DOI:** 10.1186/1472-6963-14-S2-P21

**Published:** 2014-07-07

**Authors:** Anne Cusick, Elisha Crichton, Rosalind A Bye

**Affiliations:** 1 School of Health And Society, University of Wollongong, Wollongong, NSW, Australia; 2 Occupational Therapy, University of Western Sydney, Penrith, NSW, Australia

## Background

Occupational therapists form a significant proportion of the allied health workforce in developed countries. Demand for occupational therapists in Australia is growing due to the rapidly expanding population of people living with chronic and complex conditions. Recent Australian government initiatives conducted through Health Workforce Australia have been implemented to enhance training opportunities to increase workforce supply. But increasing graduate numbers is only one part of a solution to matching workforce supply and demand. Another part that has had considerable attention in medicine but little in allied health, is where they want to work - their practice preferences.

## Methods

A survey design was used to investigate views of final year occupational therapy students. Invitations were sent to Australian universities offering occupational therapy and convenience sampling was used to recruit anonymous volunteers who completed the investigator developed survey. Survey items included: demographic data, clinical practice preference (based on [1]) and factors participants felt influenced their highest preference (based on items from [2,3]). Descriptive statistics were used to aggregate and report data.

## Results

Participants (n=259) came from every state and territory in Australia and were overwhelmingly female (91.5%; n= 236; mean age 23 years) and all were in their final year of study before graduation. Means for all clinical practice areas ranged between 51.4 and 51.8 so medians have been used to identify trends. The highest clinical practice preference was aged care (median 66, SD30.3), followed by physical rehabilitation (median 59, SD36), mental health and paediatrics (both median 54, SD16.9 and SD19.3 respectively), occupational health (median 53, SD29.8), developmental disability (median 48, SD25.8) and least preferred general physical practice (median 44, SD37).

More than half the participants thought the following factors influenced their practice preference ‘a lot’ or ‘enormously’: fieldwork experience (76.6%), fit with skills/ability(74.5%), challenge (69.4%), opportunity for greater creativity (65.8%), opportunity to perform therapy procedures (61.2%), professional development opportunities (56.4%) and intellectual content (52.3%).

## Conclusion

This study confirms previous research showing fieldwork experience is most influential in practice preference. High preference for aged care suggests good workforce demand-supply fit.

## References

[B1] Crowe MJ Mackenzie L The influence of fieldwork on the preferred future practice areas of final year occupational therapy students Australian Occupational Therapy Journal 2002 14 25 36 10.1046/j.0045-0766.2001.00276.x

[B2] Cusick A Demattia T Doyle S Factors influencing student practice preference Occupational Therapy in Mental Health 1993 14

[B3] Solomon DJ Dipette D Specialty choice among students entering the fourth year of medical school American Journal of the Medical Sciences 1994 14 10.1097/00000441-199411000-00005 7977447

